# Scalable and Controlled Production of Microalgae‐derived Extracellular Vesicles Using a Differential Tangential Flow Filtration Protocol

**DOI:** 10.1002/jex2.70185

**Published:** 2026-09-11

**Authors:** Angela Paterna, Estella Rao, Paola Gargano, Sabrina Picciotto, Giorgia Adamo, Samuele Raccosta, Antonella Bongiovanni, Mauro Manno

**Affiliations:** ^1^ Institute of Biophysics (IBF) and Cell‐Tech HUB National Research Council of Italy (CNR) Palermo Italy; ^2^ Cell‐Tech HUB and Institute for Biomedical Research and Innovation (IRIB) National Research Council of Italy (CNR) Palermo Italy; ^3^ EVE Biofactory s.r.l. Palermo Italy

**Keywords:** exosomes, extracellular vesicles, large‐scale production, tangential flow filtration, transmembrane pressure

## Abstract

The transition of extracellular vesicle (EV) production from laboratory to industrial scale presents major challenges in terms of standardization, scalability, and product consistency. Here, we present a scalable manufacturing strategy for nanoalgosomes, EVs derived from the microalga *Tetraselmis chuii*. The process integrates upstream cultivation under controlled conditions with downstream isolation and purification via continuous tangential flow filtration (TFF). Critical TFF parameters—cross‐flow velocity, shear rate, permeate flux, and transmembrane pressure—were investigated to evaluatetheir impact on process behaviour, extracellular vesicle recovery, and functional integrity. Following the MISEV2018 guidelines established in our previous baseline characterizations, the isolated nanoalgosomes were verified by a quality control, including biochemical and biophysical characterization, confirming the robustness of the platform. Overall, this work provides a practical processing framework for the scientific community dealing with large‐volume EV bioprocessing, offering an informative baseline for the scalable production of microalgae‐derived extracellular vesicles.

## Graphical abstract

This work presents a robust project design for the scalable production of extracellular vesicles, focusing on nanoalgosomes derived from microalgae. Careful parameter optimization within a filtration‐based process ensures consistent isolation while preserving the structural and functional integrity of the vesicles. The integrated workflow provides a sustainable and reproducible manufacturing platform, supported by a quality‐by‐design approach for advanced biotechnological applications.

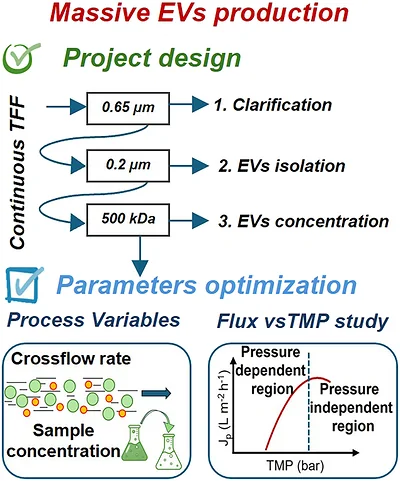

## 1 Introduction

Extracellular vesicles (EV) are defined as a heterogeneous group of particles naturally secreted by cells, characterized by a lipid bilayer, decorated with several proteins and loaded with a variety of biomolecules such as proteins, nucleic acids, and small soluble molecules (Herrmann et al. 2021; Cheng and Hill 2022). EVs, including exosomes, are identified as mediators of mcell‐cell and crosskingdom communication, resulting in physiological and pathological implications (van Niel et al. 2022). The inherent potential of EVs, stemming from their bioactive cargo or their distinct drug delivery capabilities, extends their applicability across a wide spectrum of fields. In nanomedicine, they may function as biomarkers for a range of diseases, including cancer (Möller and Lobb 2020; Urabe et al. 2020), cardiovascular disorders (Abreu et al. 2021), and immune response disorders (Xu et al. 2020) and are increasingly recognized as therapeutic and drug delivry platforms (Adamo et al. 2026; Liu et al. 2025). In cosmetics, their use is driven by their positive impact on skin health (Gargano et al. 2026; Thakur et al. 2023). In nutraceuticals, they can be used as natural components in functional foods (Qiao et al. 2022). The increasing interest among the scientific community in the significant potential of EVs as drug delivery agents has spurred their large‐scale production. Recently, the primary challenge has shifted to scaling up EV production from the laboratory to the industrial scale (Paolini et al. 2022). As a result, a wide range of alternative EV sources, particularly non‐human sources such as bacteria (Xie et al. 2023), bovine milk (Buratta et al. 2023), and plants (grapefruits, ginger, tomato, lemon) (Qiao et al. 2022; Bokka et al. 2020; Stanly et al. 2020; Urzì et al. 2023) have been explored due to their cost‐effectiveness and sustainability compared to human sources. These attributes are crucial factors in the progress of EVs from the pilot scale to the application of EV‐based products in industries (Busatto et al. 2018; Giancaterino and Boi 2023). As reported in our previous study, among 18 microalga strains, our focus was on the marine photosynthetic chlorophyte microalga *Tetraselmis chuii* (*T. chuii*) as a new sustainable biosource of small EVs, which we named nanoalgosomes (Picciotto et al. 2021). This choice was based not only on its rapid growth capabilities, which can be readily cultivated on a large scale under monitored conditions, but also on its small EVs (i.e. nanoalgosomes) production yields and quality (Adamo et al. 2021; Adamo et al. 2025). Furthermore, recent findings have shown that nanoalgosomes possess intrinsic anti‐inflammatory and antioxidant bioactivity in both tumoral and normal cell lines, as well as in *C. elegans*. They also demonstrate biocompatibility and a unique tropism for bone in mice (Adamo et al. 2024). These promising results, together with the high‐value natural components derived from microalgae, have led to the proposal of potential applications of nanoalgosomes in pharmaceutics, nutraceuticals, and cosmetics (Adamo et al. 2024; Rao et al. 2025). In general, the limitations in scaling up EV production involve both upstream and downstream processes. The upstream process involves handling large volumes of starting material, which may not always be cost‐effective and reproducible. Recently, we have optimized this process by applying batch‐refeed perfusion cultivation of *Tetraselmis chuii* cultures (Paganini et al. 2023; Picciotto et al. 2025), achieving improved quality of the *T. chuii*‐derived nanoalgosomes. The downstream process concerns the separation techniques employed to isolate them from the complex starting source. In this context, tangential flow filtration, a filtration‐based method, could potentially be scaled up for industrial‐level EV production (Giancaterino and Boi 2023; Haraszti et al. 2018; Kim et al. 2021; Su et al. 2025; Gurriaran‐Rodriguez et al. 2024; Onesti et al. 2025; Zeng et al. 2022; McNamara et al. 2018). However, although TFF has already been proposed as a scalable strategy for EV production, detailed investigations of the physicochemical and hydrodynamic processes governing continuous recirculation‐based TFF operation remain limited. Drawing on the findings of our previous investigation into the isolation of nanoalgosomes, which demonstrated a sustainable, renewable, and expandable approach (Paterna et al. 2022), we present a continuous differential TFF workflow as optimized downstream process tailored for extensive nanoalgosome separation. Here, we discuss and describe the manufacturing approach for nanoalgosome production, beginning with the scale‐up of microalgae cultures facilitated by cultivation in bioreactors in a climatic chamber (upstream processing), until high yields of concentrated sample of EVs are obtained through TFF (downstream processing). A detailed study has been conducted based on the basic principles of TFF, resulting in a set of optimized operating conditions. The main objective of this study is to explore key operational parameters that affect the process, such as the cross‐flow rate, shear rate at the membrane surface, permeate flow rate (JF) and transmembrane pressure (TMP). As already described in our previous work (Adamo et al. 2021; Paterna et al. 2022), we perform three subsequent tangential flow filtrations reducing the filter cut‐off at each step. This differential TFF, follows a sequential enrichment strategy that may be conceptually compared to differential ultracentrifugation (dUC) where subsequent sedimentation‐based separations are performed increasing the speed at each step. In the present study, the TFF‐based process is implemented as a continuous workflow, allowing a reduction in processing time while supporting reproducibility and high yields of the final product. In addition, this new downstream set‐up has been evaluated through targeted quality control checks (biochemical and biophysical characterization), for each batch of EVs produced. This characterization builds upon the extensive MISEV guidelines baseline previously established for this microalgae source (Adamo et al. 2021; Welsh et al. 2024). It also contributes to the standardization of the downstream workflow, offering a practical framework that can support the future development of scalable, GMP‐aligned manufacturing processes for small EVs (including exosomes) derived from any cell sources (Manno et al. 2025).

## 2 Materials and Methods

### 2.1 Microalgae Cultivation

A culture of the marine green algae *Tetraselmis chuii* prepared with 20‐nm ultrafiltered clear seawater. As described in our previous work (Paterna et al. 2022), the cultures were maintained for four weeks under sterile conditions, with continuous airflow, in a climatic chamber (FCC‐E‐500, Falc Instrument srl). The climate chamber was set at a constant temperature of 19±1°C and provided a mix of warm and white light with a photoperiod of 14 hours of light and 10 hours of darkness. During the growth period, the microalgal cell culture was monitored by measuring its optical density (OD).

### 2.2 TFF System

The continuous TFF system requires the use of three standalone peristaltic pumps to ensure a constant cross‐ flow rate and to support each filtration or diafiltration step. We used three Masterflex pumps with L/S 600 RPM drive with Easy‐Load II pump head (VWR), and three hollow fibers with different cutoffs and characteristics as reported in Table 1.

**TABLE 1:** Characteristics of each hollow fiber and filtered fluids involved in the TFF process.

		Step 1	Step 2	Step 3
cartridge (Cytiva) ^a^		CFP‐6‐D‐4 × 2MA	CFP‐2‐E‐4 × 2MA	UFP‐500‐E‐4 × 2MA
membrane material ^a^		Polysulfone	Polysulfone	Polysulfone
pore size ^a^ (*nm*)	*d*	650	200	20
fiber internal diameter ^a^ (*mm*)	*D*	0.75	1.00	1.00
number of fibers ^a^	*N*	75	50	50
membrane area ^a^ (*m* ^2^)	Σ	0.095	0.085	0.085
fiber length ^a^ (*mm*)	*L*	600	600	600
fluid viscosity ^b^ (*cP*)	*η*	1.240 ± 0.002	1.165 ± 0.002	1.122 ± 0.002
fluid density ^b^ (*gcm* ^−3^)	*ρ*	1.028 ± 0.003	1.027 ± 0.002	1.026 ± 0.001
characteristic flow rate ^c^ (*mLmin* ^−1^)	*Q*_0_	3.2	2.7	2.6
measured normalized water permeability (*Lm* ^−2^ *h* ^−1^ *mbar* ^−1^)	*NWP*	4.0 ± 0.1	4.8 ± 0.1	0.35 ± 0.01

^a^ Manufacturer specification.

^b^ Measured viscosities and densities for the typical fluid feeds in the three steps: (1) Microalgae culture, (2) Permeate from step 1, (3) Enriched marine water medium (f/2). The value density values are averages over different types of dilutions for each fluid.

^c^ *Q*_0_ = *NπDη*(4*ρ*)^−1^: characteristic parameter to determine the Reynold number *R_e_* from the operational flow rate *Q_F_*, so that: *R_e_* = *Q_F_* / *Q*_0._

**FIGURE 1:**
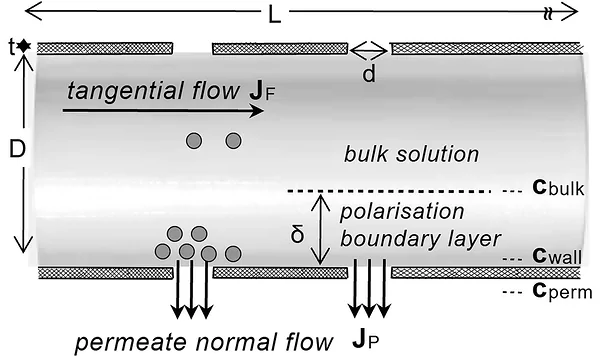
Schematic representation of TFF systems, displaying the tangential and normal fluxes (**J** *_F_* and **J** *_P_*, respectively), the thickness δ of the concentration polarisation boundary layer, the relevant boundary layer concentrations for a solute in the flow **c** *_bulk_*, **c** *_wall_* and **c** *_perm_*, that are the bulk concentration, the concentration at the membrane wall and the concentration of the permeate solution, respectively.

Pressure sensors (1/4″ hose barb inlet/outlet, PendoTECH) equipped with a pressure monitor (PendoTECH, PMAT3‐BAR PRESSURE‐ MATTM) are placed to measure the inlet pressure in the feed, retentate, and permeate lines. A precision peristaltic tube (Peroxide‐cured silicone tubing, L/S 25, i.d. 4.8 mm, max flow rate 1000 mL min^−1^, MFLX96400‐ 25, VWR) is used to connect the feed and retentate ports (12.7 mm Tri‐Clamp) as well as the permeate port (9.5 mm tubing nipple), via female luer threads (1/4″ ID tubing, Spectrum Lab), to GL45 screw connection caps equipped with three GL14 ports, fitted to the sterile holder unit. The empty sterile bottles connected to the permeate lines are placed on a scale (VWR, Premium Balance Precision) to measure the permeate weight and assess the flux achieved during each filtration process (Figure 1). Pressure data and permeate flux (LMH, liters per hour per square meter) are recorded manually every two minutes until the desired transmembrane pressure (TMP) and flux are reached; then, they are recorded every five minutes during the entire process. The entire system operates within a refrigerator (1400 L, JKS Refrigeration S.r.l.) set at 8°C to carefully regulate the temperature and prevent any degradation of the samples. To obtain an enriched sample of small EVs in PBS, a 500 kDa polysulfone hollow fiber cartridge (UFP‐500‐C‐MM01A, Cytiva) has been used to concentrate and diafiltrate the preparation of EVs.

### 2.3 Extracellular Vesicles Characterization

#### 2.3.1 DLS

A defined volume of the vesicle suspension was centrifuged at 1000×g for 10 minutes at 4°C to eliminate potential particulate contaminants. The resulting supernatant was carefully transferred, using MilliQ‐rinsed pipette tips, to a clean quartz cuvette and equilibrated at 20°C within the thermostated chamber of a BI200‐SM goniometer (Brookhaven Instruments). The instrument was configured with a He‐Ne laser source (*λ* = 633 nm, JDS Uniphase 1136P) and a single‐photon counting detector (Hamamatsu C11202‐050). Dynamic light scattering measurements were carried out by ac‐ quiring the intensity autocorrelation function *g_2_(t)* at a fixed scattering angle of *θ* = 90°, using a digital BI‐ 9000 correlator (Brookhaven Instruments). The scattered intensity and its temporal correlation provide insight into the vesicle size distribution *P_q_(σ)* through the relation: $g_{2}\;\left(t\right)=1+\beta |\int P_{q}\left(\sigma \right)\exp\left\{-D\left(\sigma \right)q^{2}t\right\}d\sigma {|}^{2}$, where *β* is the coherence factor of the detection system, *q  =  (4πñ/λ) sin(θ/2)* represents the magnitude of the scattering vector, with *ñ  =  1.3367* being the refractive index of the solvent. The diffusion coefficient *D(σ)* corresponding to vesicles of hydrodynamic diameter was calculated using the Stokes‐Einstein relation: *D(σ)  =  k_B_T/*(3*πησ*), in which *k_B_* is Boltzmann's constant, *T* is the absolute temperature, and *η* the dynamic viscosity of the dispersion medium (Berne and Pecora 1990). The function *P_q_(σ)* was reconstructed under the assumption that the underlying distribution of the diffusion coefficient is the Schultz distribution, an asymmetric model of two parameters defined by a mean diffusion coefficient* ⟨D⟩* and a variance V  =  ⟨[D‐⟨D⟩]^2^ (Paterna et al. 2022; Mailer et al. 2015; Noto et al. 2012). This approach offers robustness against experimental noise and enables the extraction of key physical descriptors, namely: ⟨σ⟩, the z‐average hydro‐ dynamic diameter, PDI, the polydispersity index, calculated as PDI  =  V ⟨D⟩ ^−2^, which quantifies the breadth of the size distribution.

#### 2.3.2 NTA

The concentration and size distribution of the nanoparticles were evaluated using a NanoSight NS300 (Malvern Panalytical, UK). EVs were diluted in HPLC‐ grade water (Sigma‐Aldrich) previously filtered by 20 nm filters (Whatman Anotop) to obtain 20–120 particles per frame. Five 60‐second videos per sample were recorded in light scattering mode at a syringe speed of 60, with camera settings adjusted to level 15–16. The recorded data was processed using NanoSight Software NTA 3.1 Build 3.1.46, applying the appropriate detection threshold.

#### 2.3.3 AFM

Atomic Force Microscopy (AFM) analysis was carried out using a Nanowizard III scanning probe microscope (JPK Instruments), configured with a 15 *µ*m z‐range scanner. The nanoalgosome preparations were first concentrated by ultracentrifugation and subsequently resuspended in MilliQ grade water to a final particle concentration of 10^11^particles/mL, as quantified by nanoparticle tracking analysis (NTA). For sample deposition, 30 *µ*L of the suspension was applied to freshly cleaved mica substrates and allowed to adsorb for 10 minutes at ambient conditions, followed by gentle drying under a nitrogen stream. The imaging was performed in tapping mode employing an NSC‐15 cantilever (Mikromasch), characterized by a nominal spring constant of 40 N/m and a tip radius of 8 nm. 5 × 5*µ*m^2^ and 500 × 500 nm^2^ images were acquired at 512 × 512 and 256 × 256 pixel resolution, respectively. Setpoint was fixed at 70% of free oscillation amplitude (20 nm).

#### 2.3.4 BCA

The Pierce BCA Protein Assay Kit (Thermo Fisher Scientific) was used to determine the protein concentration of the nanoalgosomes, following the manufacturer's instructions. Briefly, nanoalgosome samples and bovine serum albumin (BSA) standards (0‐200 µg mL^−1^) were loaded into a 96‐well microplate and mixed with the working reagent solution prepared according to the kit protocol. Samples were incubated at 37°C for 30 min to allow the colorimetric reaction to proceed. Subsequently, the absorbance of the BCA protein complex was measured at 560 nm using a Bio‐Rad iMark microplate absorbance reader. Protein concentration was calculated from the BSA standard calibration curve and expressed as µg mL^−1^.

#### 2.3.5 Western Blot

The separation of nanoalgosome proteins and microalgal lysate was performed using sodium dodecylsulfate polyacrylamide gel electrophoresis (SDS‐ PAGE) (10%). 2X or 4X SDS sample loading buffer (0.2‐0.1 M Tris‐Cl pH 6.5, 4%–8% (w/v) SDS, 20%–40% glycerol 4.3 M, 2%–4% bromophenol blue, 10%–20% 2‐ mercaptoethanol) was added to 15 *µ*g of EVs in Dulbecco's PBS or 20 *µ*g of microalgae lysate in RIPA lysis buffer. The samples were denatured at 100°C for 7 minutes, loaded onto SDS‐PAGE, and run at 100 V. The proteins were then transferred onto polyvinilidene fluoride membranes (PVDFs), which had been preactivated with methanol and washed with water and transfer buffer. The membrane was incubated overnight at 4°C with the antibody anti H+‐ATPase (diluted 1:1000 in blocking buffer diluted 1:1 with TBS/Tween 0.2%, Agrisera AS07 260; Polyclonal; Host: Rabbit), or anti α‐Tubulin antibody (diluted 1:1000 in blocking buffer diluted 1:1 with TBS/Tween 0.2%, Sigma‐Aldrich T5168; mouse monoclonal, B‐5‐1‐2). The membrane was washed and incubated with goat anti‐rabbit secondary antibody (dil 1: 5000 in blocking buffer diluted 1:1 with TBS/Tween 0.1%, LICOR IRDye 680RD) for 1 hour at room temperature. Protein bands were detected using the Odyssey DLx Imager and its associated software (LI‐COR, USA).

#### 2.3.6 Size Exclusion Chromatography (SEC)

High performance size exclusion chromatography (HPSEC) was performed to evaluate EVs purity. The purification was carried out using a column (XK 16/20 Cytiva; internal diameter 16 mm, length 20 cm) manually packed with Sepharose CL‐2B (bed height 18 cm), connected to Vanquish UHPLC System, Thermo Scientific Ultra High Performance Liquid Chromatography (UHPLC) system equipped with quaternary pump (VC‐P20‐A‐01), split loop autosampler (VC‐A13‐A‐ 02), UV‐vis photodiode array detector (VC‐D11‐A‐01), fluorescence detector D‐PMT (VC‐D51‐A‐01), integral fraction collector (VF‐F20‐A‐01) and Postnova PN3621 Multiangle Light Scattering Detector. After column equilibration with Dulbecco's PBS, an isocratic separation was achieved using the same mobile phase. 200 *µ*L of diluted nanoalgosomes stained with 500 *n*M of 4‐(2‐[6‐(dioctylamino)‐2‐ naphthalenyl]ethenyl)‐1‐(3‐sulfopropyl)pyridinium, Di‐8‐ANEPPS for 1 hour at room temperature has been injected applying a flow rate of 1 mL min^−1^. The Optical Density (OD) spectra were acquired in the UV‐visible range, and fluorescence spectra (*λ_ex_* = 488 nm) were acquired and fractions of 500 *µ*L were collected.

## 3 Results and Discussion

### 3.1 Upstream Process Scale‐up

In our previous work, we detailed the establishment of a permanent sterile platform for the cultivation of microalgae on the pilot scale 35]. To maintain constant and reproducible microalgae cell growth and vitality, the lab has been equipped with a 500 L climatic chamber. Microalgae cultures are grown for four weeks under continuous airflow and in sterile conditions. The climatic chamber allows us to maintain the temperature at 19±1°C and ensures exposure to a mixture of warm and white light with a photoperiod of 14 hours of light and 10 hours of darkness. Under these conditions, we can obtain 60 L of microalgae culture per month, that can be used as starting material for downstream EV isolation. Further optimization of large‐scale cultivation parameters is described in our recent work (Picciotto et al. 2025).

### 3.2 Downstream Process Scale‐Up

#### 3.2.1 TFF Operating Principles and Parameters

Tangential flow filtration (TFF) was used as a gentle methodology to process large volumes of microalgae conditioned media while preserving nanoalgosome integrity. Because the systematic design and operation of a TFF system relies on the evaluation of multiple interconnected variables (Figure 1), an operational framework balancing fluid dynamics and mass transfer characteristics was implemented. This approach allowed for the definition of a stable operating window without requiring exhaustive empirical testing. In this section, we briefly define the main TFF parameters, while the underlying fluid dynamics and mass transfer governing equationsare detailed in Supplementary Note 1.

The **crossflow flux** also known as the feed flux or recirculate flux *J_F_* is defined as the volumetric flow rate *Q_F_* through the channel per unit area *A*. Based on the physical dimensions of our hollow fiber cartridges and fluid characteristics, our system constants reveal that the operational crossflow volumetric flow rates (*Q_F_*) maintain the process strictly within the laminar flow regime (low Reynold number: *Re*≤2300, see Table 1) (Mailer et al. 2015; Noto et al. 2012; Landau and Lifshitz 1987), and limit the shear rate at the membrane wall, that may affect the the quality of the final product.

Along with the tangential crossflow, in TFF systems the solution is selectively driven through the filtration membrane. This so‐called **permeate flux** *J_P_*, defined as the volumetric flow rate Q*_P_* through the membrane per unit area *Σ_m_*, is associated with the **transmembrane pressure** ∆*P_TMP_*, also known as TMP in the technical literature. In laboratory practice, under the assumption that the pressure inside the flow channel is the average between the pressures at the two ends (feed and retentate pressures), which is of course an approximation, this pressure difference is calculated as follows:

(1) $$\Delta \;P_{TMP}=\frac{P_{feed}+P_{retentate}}{2}\;-P_{permeate}\equiv TMP$$

For water, or simple fluids, the permeate flux *J_P_* is directly proportional to the transmembrane pressure ∆*P_TMP_* with a coefficient called membrane permeability (Lutz 2010). In particular, **normalized water permeability** (*NWP*) is a parameter that is often used to determine the efficacy of the cleaning process and the integrity of the filter. *NWP* is measured after cleaning and is compared to the value measured at membrane installation. In order to be independent of the operating temperature NWP is defined using a correction factor (*TCF*) that reports the viscosity of the water at a certain temperature *η(T)* to the viscosity of the water at 20°C:

(2) $$NWP=\frac{J_{P}}{\Delta P_{TMP}}\;\frac{\eta \left(T\right)}{\eta \left(20\right)}\;\left[NWP=\frac{LMH\times TFC}{TMP}\;\right]$$

Since TFF operates to transfer solute particles across the membrane, one may consider that the transmembrane pressure operates against different resistances to the flow:

(3) $$\Delta \;P_{TMP}=\left(R_{m}+R_{bl}+R_{f}\right)\eta J_{P}$$

The main effects are (i) a resistance due to the **membrane R*_m_*** (Field 2010), that is the reciprocal of the permeability; (ii) a resistance due to the **boundary layer R*_bl_*** (Asmolov 1999; Zydney 1997; Wijmans et al. 1985), that is the the region close to the membrane where a concentration gradient is established due to diffusion along the radial direction of the channel; (iii) a resistance due to **membrane fouling R*_f_*** (Landau and Lifshitz 1987; Field et al. 1995), caused by the crowding of particles at the membrane walls.

**FIGURE 2:**
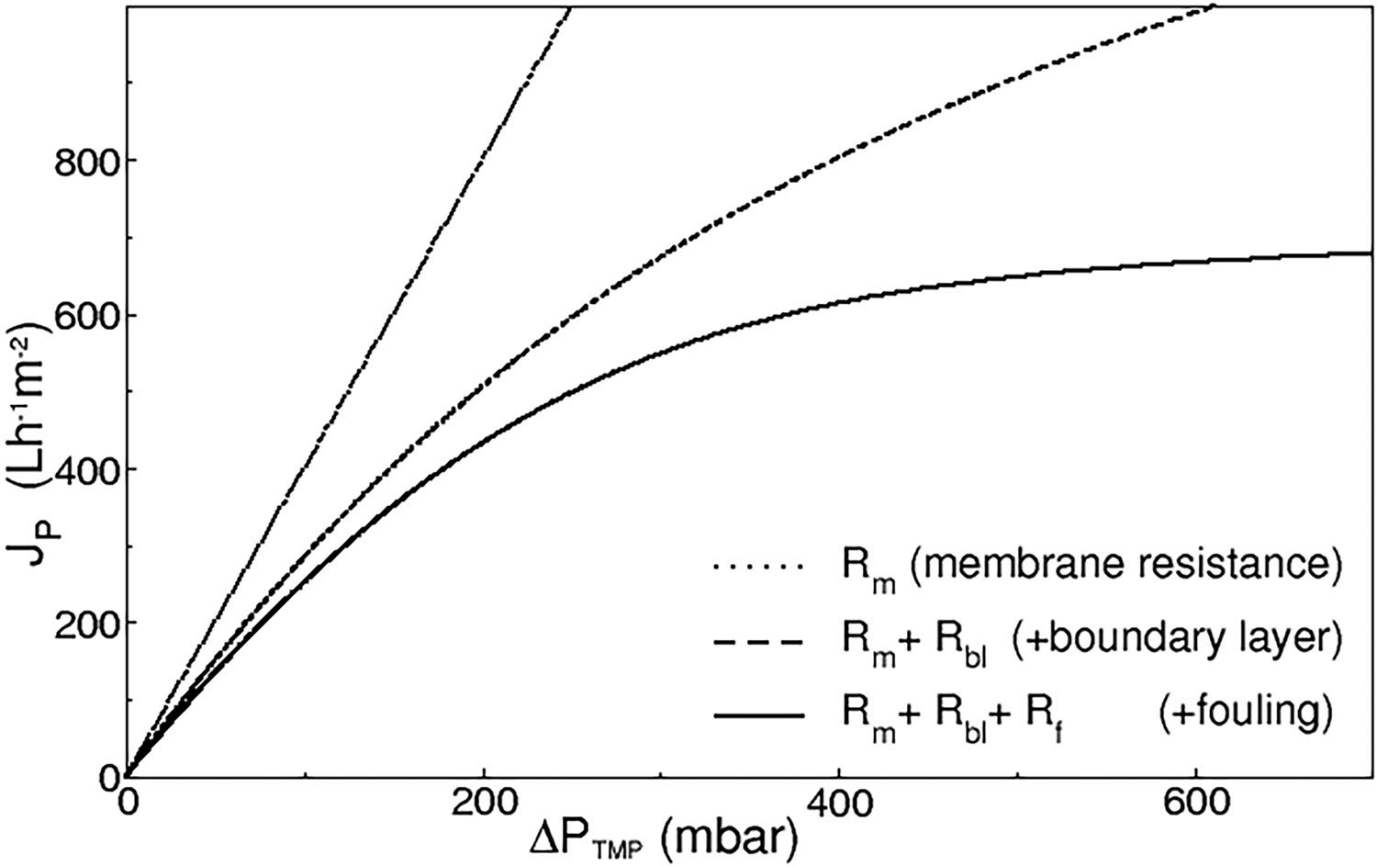
Permeate flux **J** *_P_* versus transmembrane pressure ∆**P**_TMP_ forgiven values of membrane resistance R*_m_*, boundary layer resistance R_bl_ and fouling resistance R*_f_*; (i) dotted line: R*_m_* = 0.25 mbar (L m^−2^ h^−1^)^−1^, R*_bl_* =0, R*_f_* =0; (ii) dashed line: R*_m_* as in the previous curve, R*_f_* =0, R*_bl_* determined assuming a bulk osmotic pressure *π*_0_= 24 mbar and a mass transfer coefficient κ= 360 L m^−2^ h^−1^ = 100 µm s^−1^; (iii) solid line: R*_m_* and R*_f_* as in the previous curve, R*_f_* determined assum‐ing a threshold flow *J*_1_= 720 L m^−2^ h^−1^ = 200 µm s^−1^ and a pressure *π*_1_ = 24 mbar.

At low flux values, the main resistance is due to the membrane (linear regime), while at higher flux values the flow is affected by the boundary layer (polarisation regime) (Lutz 2010; Wijmans et al. 1985); eventually, fouling becomes relevant, rate‐limiting and it affects the transmembrane pressure (Wijmans et al. 1985; Kirschner et al. 2019). Figure 2 illustrates the effects of different resistances by using the derived expressions for membrane and boundary layer resistances and an ad hoc expression to model fouling resistance (see Supplementary Note 1).

It is important to mitigate or avoid membrane fouling in order to improve filtration efficiency. A direct method to remove fouling could be to induce a turbulent flow regime and thus to reduce the concentration boundary layer. Other methods include periodic back wash cleaning during the filtration process and not only for cleaning and recovery (Romann et al. 2024). Here, we adopt a different strategy that is to limit the flux and transmembrane pressure to the first polarisation regime, avoiding both the gel layer formation and the turbulence. In this manner, we have the highest possible flux at low Reynolds number, that is, in the laminar flow regime, avoiding a large shear stress that may affect our products. This is relevant not only for the isolation of extracellular vesicles itself, but also for the clarification step and to preserve the integrity of the cell sources, since we demonstrated the possibility of recycling the cell culture for the subsequent production of EV (Paterna et al. 2022).

### 3.3 Process Design and Optimization of Operational Parameters

The isolation of EVs from microalgae is carried out through a multistep process that involves two microfiltration hollow fibers (cutoff 0.65 and 0.2 *µ*m), and one ultrafiltration hollow fiber (cutoff 500 kDa). The surface area of the selected membranes enabled the processing of up to 10 L of solution within a single run under the applied operating conditions, primarily influencing the processing time required to handle this volume. Each process step (or TFF module) has a different specific purpose (Figure 5):

- *Clarification*: 0.65 *µ*m polysulfone hollow fiber cartridge; microalgae harvesting consists in separating microalgae biomass from all the smaller particles present in the medium. In this step, intact microalgal cells were retained, while the permeate was forwarded to the subsequent filtration stage.
- *Isolation*: 0.2 *µ*m polysulfone hollow fiber cartridge; the permeate resulted from the clarification is the starting solution of this cartridge (particle size < 0.65 *µ*m); in this step the retentate obtained is enriched sample of particles ranging from 0.65 to 0.2 *µ*m (large vesicles); This fraction was not further characterized in the present study because previous analyses indicated the presence of negligible EV‐associated material under the adopted experimental conditions. Nevertheless, larger extracellular vesicle populations released by microalgae have been previously isolated and characterized in our earlier work (Paterna et al. 2022).
- *Ultrafiltration and concentration*: 500 kDa polysulfone hollow fiber cartridge; the permeate of the second cartridge is ultrafiltered (approximate equivalent hydrodynamic size 20 nm based on MWCO calibration) removing protein and all small particles presents in the medium and concentrated (small vesicles). This step was employed to concentrate small EVs while allowing soluble proteins and other low‐molecular‐weight components to pass into the permeate.
- *Diafiltration and volume reduction*: In the final step, a 500 kDa hollow fiber cartridge with a reduced internal volume was used to further concentrate the sample to a final volume of approximately 5 mL and, when required, to perform buffer exchange by diafiltration. In the present study, diafiltration was performed using PBS as exchange buffer.

The closed TFF‐based configuration enables processing under sterile conditions, minimizing the risk of contamination throughout the entire process.

#### 3.3.1 Pre‐Processing: Water Permeability Test

**FIGURE 3:**
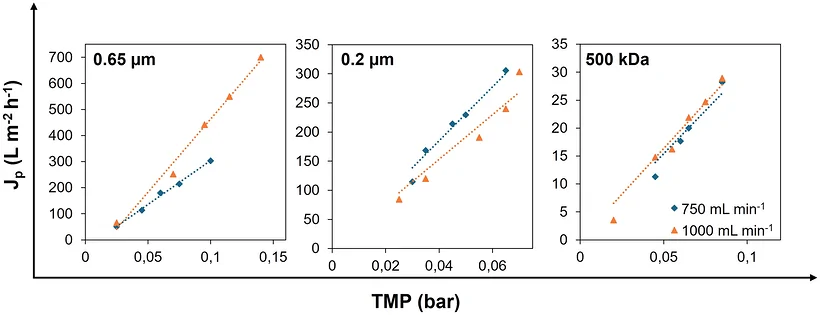
Water permeability test. *J_P_* vs ∆*P_TMP_* curves at constant crossflow rate (750 or 1000 mL min^−1^). Different ∆*P_TMP_* values are obtained by clamping the retentate tube.

Before starting the isolation protocol, we conducted a thorough evaluation of the water permeability of each module at various TMP. This experiment was designed to ensure that the modules would perform optimally under different pressure conditions, which is crucial for the subsequent concentration process. To perform the water permeability test, MilliQ water was flushed at two different crossflow rates (750 and 1000 mL min^−1^) and the transmembrane pressure ∆*P_TMP_* was changed by clamping the retentate tube. In Figure 3 the plot of the permeate flux *J_P_* versus ∆*P_TMP_* shows a linear behaviour, which indicates that the module is clean and intact. Subsequently, an initial value of the normalised water permeability NWP was calculated for each module to establish a starting point before processing any samples. To minimize variability and have a most reliable value, the NWP is measured at fixed crossflow rate and TMP.

#### 3.3.2 Processing: ∆*P_TMP_* Scouting

**FIGURE 4:**
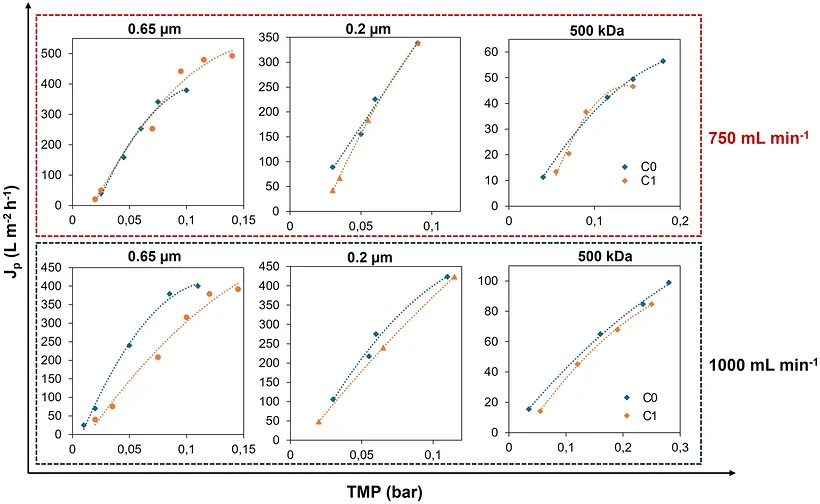
*J_P_* vs ∆*P_TMP_* curves at constant crossflow rate (Upper panels: 750 mL min^−1^; Lower panels: 1000 mL min^−1^).

To determine the optimal filtration conditions for each cartridge involved in the process and to prevent the formation of a gel layer on the membrane surface, thus maintaining membrane performance over time, a TMP scouting procedure was performed. The crossflow rate was kept constant and permeate flux *J_P_* versus transmembrane pressure ∆*P_TMP_* curves were generated for each membrane. A peristaltic pump controlled the crossflow rate. In addition, to understand the behavior of the filtration process, experiments were conducted at two different crossflow rates and two different feed fluid concentrations. For this purpose, a bioreactor containing 6 L of *T. chuii* culture, consisting of 4.3 × 10^5^ cells mL^−1^and an OD of 0.44 cm^−1^ has been divided into four samples to evaluate the best parameters for each membrane at two different solution concentrations and two different crossflow rates. In particular, two samples (labelled as C0 in Figure 4) represent the microalgal culture at its final growth stage (OD 0.44 cm^−1^, 4.3 × 10^5^ cells mL^−1^) and two samples (labelled as C1 in Figure 4) represent the culture diluted twice (OD 0.22 cm^−1^, 2 × 10^5^ cells mL^−1^). It should be noted that, within the integrated continuous workflow, only the first membrane module (0.65 µm) directly processes the complete microalgal suspension containing intact cells and larger particulate matter. The subsequent membrane modules operate sequentially on the permeate generated by the previous filtration step and therefore process progressively clarified solutions with different physicochemical characteristics. Accordingly, the C0 and C1 conditions were employed as preliminary screening conditions to investigate the effect of feed concentration and hydrodynamic operating parameters on membrane performance under standardized and comparable experimental settings. To evaluate the variation in flux as a function of ∆*P_TMP_*, high transmembrane pressures were achieved by placing clamps to the retentate and permeate tubing, after the retentate and permeate sensors. As shown in Figure 4, the generation of curves *J_P_* versus ∆*P_TMP_* allowed us to identify different regions dependent on TMP. The graphs illustrate how the flux increases with increasing TMP until the effects of concentration polarisation reduce any further increase of *J_P_*. We are still not reaching a plateau, which indicates the formation of a gel layer, but a loss of efficacy is clearly evident at higher TMP values. These experiments allow us to select the optimal working conditions for the three cartridges used in the EV isolation and purification process.

The process of isolating EVs from microalgae in our previous work was described as renewable, involving the recycling of microalgae cells after TFF isolation for multiple cycles of EVs production (Paterna et al. 2022). In that study, microalgal viability following the initial TFF step using a 0.65 µm cartridge was evaluated to assess potential adverse effects of filtration on cell growth. Cell viability was assessed indirectly by monitoring microalgal growth over a four‐week cultivation period following filtration, using OD measurements as an indicator of cell proliferation. Under the tested cross‐flow conditions, no appreciable differences in growth trends were observed compared to non‐processed cultures, suggesting that the clarification step did not adversely affect cell viability. Furthermore, since there were no significant differences in the yield of EVs and the growth of microalgae cells, the setup chosen for the entire process is 750 mL min^−1^. As shown in Figure 4, variations in microalgae concentration do not significantly impact the isolation process, highlighting that sample dilution is not necessary prior to sample processing.

For each membrane, two crossflow rates were initially evaluated during TMP scouting to generate permeate flux–transmembrane pressure (JP–ΔPTMP) profiles and assess process behaviour under different hydrodynamic conditions. These experiments were not intended to define multiple optimal operating points but to identify a single robust operating window suitable for continuous processing. Based on these results, a final operating condition was selected for each membrane module (750 mL min^−^^1^ crossflow rate), which ensured stable operation, reproducibility, and compatibility across the integrated TFF workflow. The corresponding TMP values reported in Table 2 reflect the operating pressures achieved under these final set conditions.

**TABLE 2:** Final operating conditions selected for each hollow‐fiber membrane module following TMP scouting experiments.

Set‐up parameters		Process variables		
**Pore size**	**Crossflow rate** (*mL min* ^−1^)	**Shear rate** (*s* ^−1^)	**LMH** (*L m* ^−2^ *h* ^−1^)	**TMP** (*bar*)
0.650 *µm*	750	4021	150	0.045
0.200 *µm*	750	1187	200	0.050
500 *kDa*	750	2546	37	0.090

#### 3.3.3 Post‐Processing: Cleaning and Storage

At the end of each experiment, the cartridges undergo a flushing process with 2 L of MilliQ water to remove residual sample deposits from the membrane surface. This is followed by a cleaning step using 0.5 M sodium hydroxide (NaOH) at 50°C for 30 minutes at a flow rate of 1000 mL min^−1^ in recirculation mode. After this initial cleaning phase, the permeate line is fully closed, allowing the cleaning solution to remain in contact with the membrane for an additional 60 minutes. Subsequently, MilliQ water is used to flush out the NaOH solution until a neutral pH is achieved, ensuring membrane restoration. Finally, the normalized water permeability (NWP) is calculated to assess cartridge condition after cleaning. If the NWP drops by more than 20% with respect to the initial value, it is possible that the cleaning cycle was not successful and another cleaning cycle should be performed. After the cleaning procedure, each module is dried and stored at 4°C until its next use. In addition, tubing, sensors, and fittings are cleaned with 70% ethanol followed by MilliQ water. In a GMP‐regulated bioprocess, this post‐processing step must be adapted to comply with regulations by strictly using a new, single‐use cartridge for each batch, as reuse is not permitted.

#### 3.3.4 New Continuous TFF System Configuration

**FIGURE 5:**
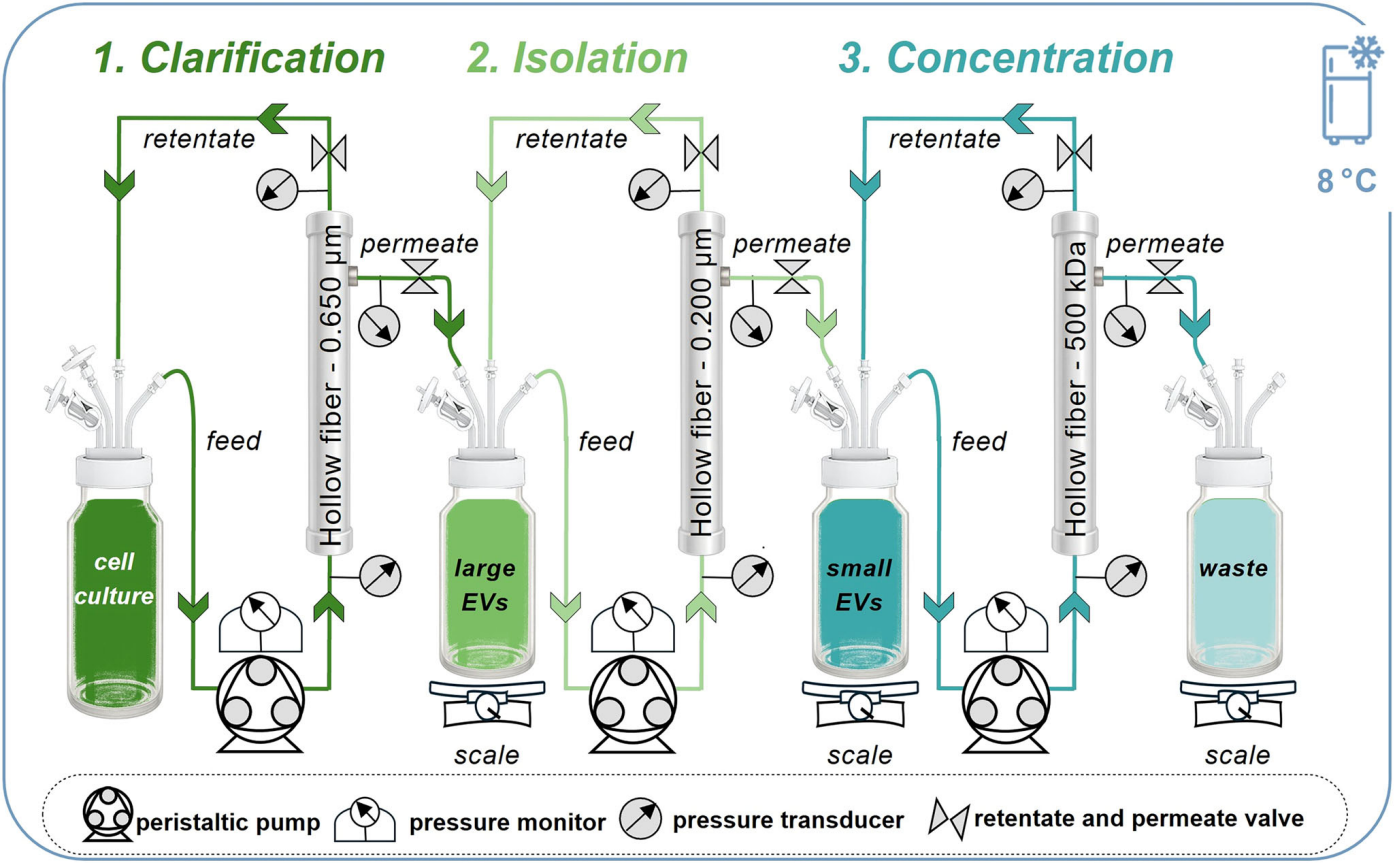
Schematic flow diagram of the new TFF continuous system for isolation of extracellular vesicles from cell culture. The system is designed with a three‐stage configuration, where each stage includes an individual peristaltic pump, pressure sensors and monitor, and scale (left to right). The entire process temperature is kept constant at 8°C.

Figure 5 describes the assembly of the TFF system for conducting a continuous TFF process to isolate nanoalgosomes from microalgae under sterile conditions and with temperature control. The system requires three empty 2 L sterile bottles and one holder containing the sample, all connected to the TFF system through GL45 screw connection caps with three GL14 ports. Each port is connected to the precision peristaltic tube using a female luer thread (1/4″ ID tubing, Spectrum Lab). The empty sterile bottles are placed on a scale to evaluate the flux achieved in each filtration process Afterwards, in normal operating procedure the three steps are carried out continuously; in such a case the scales are not strictly necessary and the pumps are working in series decoupled by the feed bottle for each step. To maintain sterility, the peristaltic tube is cleaned with a 70% ethanol solution before use, and the entire system is flushed with MilliQ water. The pressure monitor is located above the peristaltic pump and pressure sensors are installed for pressure measurements in the feed, retentate, and permeate lines of each cartridge. It is essential to monitor flux at a specified TMP throughout the filtration process because permeate flux decreases with increasing concentration. Consequently, as the feed concentration increases, higher TMPs are required to maintain the same permeate flux. The clamping of the retentate tube is necessary to achieve the desired permeate flux rate. Figure 5 illustrates the continuous TFF process in three stages starting from 6 L of *T. chuii* culture: an initial filtration through a 0.65 µm cartridge, achieving a 20‐fold concentration after the first stage; a secondary filtration through a 0.2 µm cartridge, reaching a 30‐fold concentration; and a final concentration step using a 500 kDa module, attaining an overall 40‐fold concentration. The three pumps are set at 750 mL min^−1^. The process begins by closing all the permeate lines and starting the first pump in recirculation mode until a stable TMP is established. Slowly, the permeate of the first cartridge is fully opened, reaching a ∆*P_TMP_* of 0.045 bar and a *J_P_* of 150 L m^−2^h^−1^. During the entire filtration in the first step, the concentration may increase significantly. Therefore, it is crucial to clamp the retentate tube to achieve the target permeate flux. Once the first permeate container reaches one litre, the second pump is activated. After stabilization of ∆*P_TMP_* in the recirculation mode, the permeate is gradually released, reaching a ∆*P_TMP_* of 0.05 bar and a *J_P_* of 200 L m^−2^h^−1^. The third pump is turned on once the second permeate holder contains a volume greater than one litre of filtered sample. When the TMP is constant, the permeate is opened, attaining a ∆*P_TMP_* of 0.09 bar and a *J_P_* of 37 L m^−2^h^−1^ prompting the start of the final concentration phase.

#### 3.3.5 Diafiltration

Eventually, an additional concentration step may be added by using a 500 kDa cartridge with a smaller volume (C02‐E500‐05‐N), to reach a final solution of 5 mL. In the present work, the UF/DF step was performed using the 500 kDa module operated at a crossflow rate of 75 mL min^−1^, a *J_P_* of 23 L m^−2^h^−1^, and a *∆P_TMP_* of 0.25 bar. The sample volume was first concentrated from 150 to 15 mL, followed by diafiltration with 150 mL of Dulbecco's PBS under constant‐volume conditions, and subsequently reconcentrated to a final volume of 5 mL. This step may also be used for efficient and fast diafiltration and buffer exchange, as described above. A typical buffer exchange procedure is performed by subsequent steps, for instance by centrifugation, in which the initial volume *V_0_* is reduced by a volume ∆*V* with a relative volume reduction *x  =  ∆V/V_0_* and a concentration increase with respect to the initial concentration *c_0_* of *c*/*c_0_*  =  (1−*x*)^−1^; then the residual fraction of the old solvent is *f * =  1−*x*. After *N* steps this residual fraction is *f*  =  (1−*x*)*^N^*, but in each step the solute concentration has been raised by a factor of *(1−x)^−1^*, with possible risks related to solute instability. In diafiltration, the relative volume reduction (*x*) is infinitesimal, and the addition of *n* column volumes is equivalent to a number of steps *N* = *n*/*x*. In the limit of *x* going to zero one obtains: $c/\;c_{0}=lim_{x\to 0}\;{\left(1-x\right)}^{-1}=1$ and $f=lim_{x\to 0}\;{\left(1-x\right)}^{-n/x}=e^{-n}$. Thus, the solute con‐ centration remains constant and the old buffer residual decreases exponentially with respect to the number of volumes exchanged. For example, after *n * =  7 circulating volumes, the residual old buffer is *f*  =  0.000912, that is, less than 0.1%.

### 3.4 Nanoalgosomes Biochemical and Biophysical Characterization

Quality control checks have been conducted on at least three batches of nanoalgosome production, starting from 6 L of microalgae culture, to ensure the reproducibility of the new continuous isolation system. Nanoalgosomes have been extensively characterised in our previous work (Picciotto et al. 2021; Adamo et al. 2021; Paterna et al. 2022), as outlined in the MISEV guidelines (Welsh et al. 2024; Witwer et al. 2017); here we perform the basic routinary biochemical and biophysical quality check analyses on nanoalgosome batches.

**FIGURE 6:**
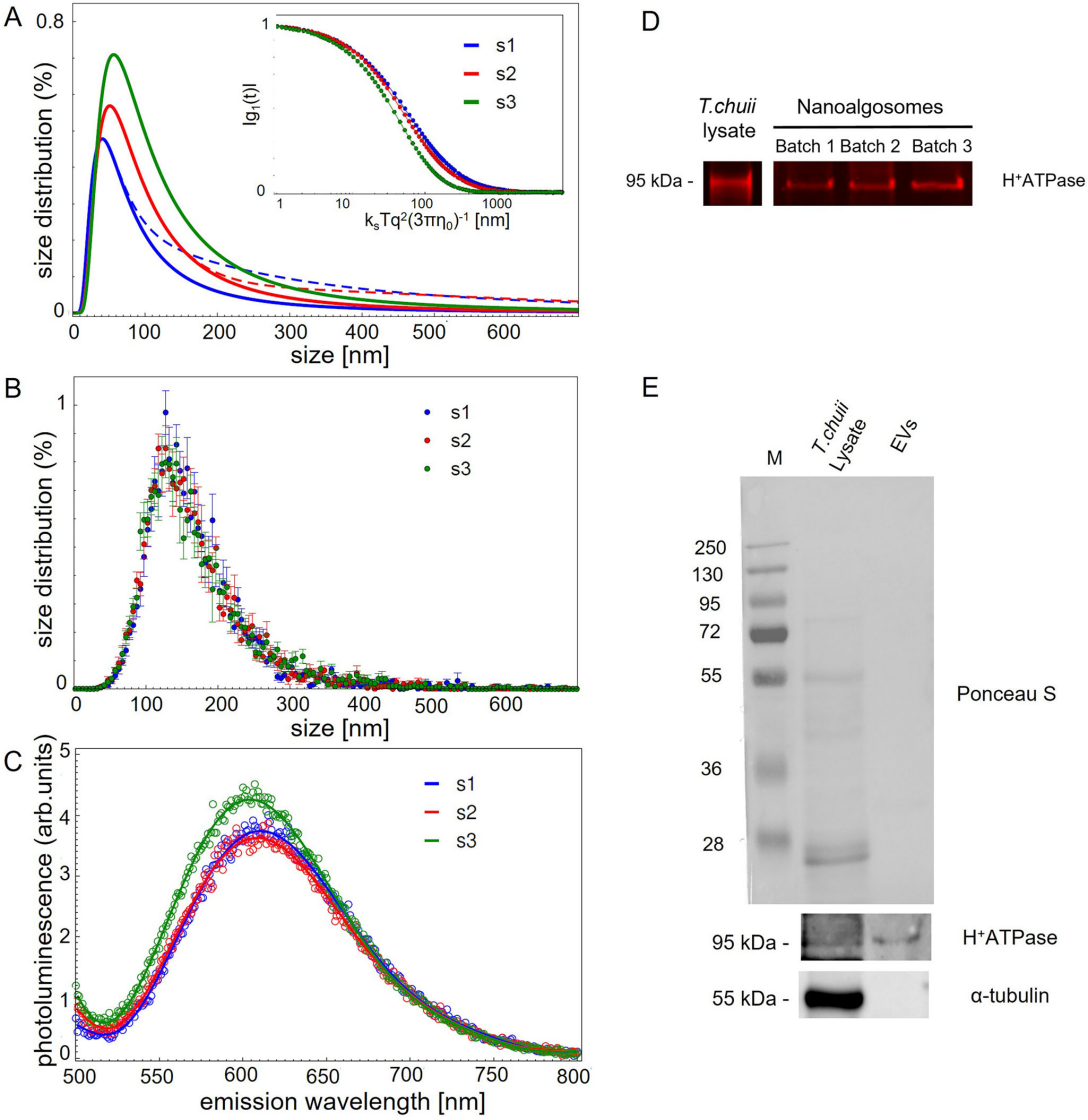
EV post‐processing characterisation for 3 independent batches (s1, s2, s3). A. Light scattering analysis: size distribution functions, derived from the measured intensity autocorrelation functions (inset); B. Nanoparticle Tracking Analysis: size distributions; C. Photoluminescence Spectra of EVs labeled with Di‐8‐ANEPPS fluorescent dye (with excitation wavelength of *λ_ex_* =488 nm); D. Western blot of H+‐ATPase for the 3 EVs batches with respect to the cells lysate; E. Western blot of H+‐ATPase and of α‐tubulin as negative control for nanoalgosomes with respect to the cells lysate.

*Size distribution and concentration*. The size distribution was measured by dynamic light scattering (DLS) and nanoparticle tracking analysis (NTA). In particular, DLS can determine the entire size distribution in a sample of nanoparticles from a few nanometers to hundreds of nanometers, thus eliciting whether the sample also contains small molecules or large aggregates along with the expected population of EVs (Adamo et al. 2021). We analyze the intensity autocorrelation functions (inset of Figure 6A) using the Shultz distribution for the diffusion coefficient (Berne and Pecora 1990), that is, a two‐parameter distribution, whose skewness and, in general, all high‐order cumulants are determined uniquely by the average ⟨σ⟩ and the variance This minimalistic approach, admitted by the typical noise level and in the experimental auto correlation functions (Mailer et al. 2015), is a key point for deriving useful and readable information from DLS experiments, better than the classical regularization methods (Noto et al. 2012). In comparison, NTA is less definitive in determining the actual size distribution (Figure 6B), as it is more accurate in measuring large particles. Nevertheless, NTA remains very useful to obtain a direct extimate of particle concentration (Adamo et al. 2021). Another method of determining the amount of nanoparticles is to measure their total protein content; we used the commonly used BCA colorimetric assays. A summary in Table 3.

**TABLE 3:** Size and concentration parameters. The errors are calculated as the standard deviations for the measurements done in three independent batches.

DLS hydrodynamic diameter ⟨*σ*⟩ (nm)	70	±	2
DLS polydispersity index *PDI*	0.329	±	002
NTA particle concentration (EVs mL^−1^)	1.4 × 10^12^	±	0.1 × 10^12^
BCA protein content (*µ*g mL^−1^)	93	±	11

Overall, characterization measurements consistently demonstrated reproducible yields across the three EV batches (∼10^12^ nanoalgosomes L^−1^ of microalgal conditioned medium), corresponding to ∼ 94 µg L^−1^of microalgae culture, with a particle protein ratio ∼10^10^ µg^−1^ as in ref. (Paterna et al. 2022), thus confirming the robust reproducibility of the production process.

*Lipid bilayer and protein markers*. Furthermore, the identity of the EV and the consistency of the lipid bilayer have been assessed by (i) labelling the EV membrane with a specific lipophilic dye, Di‐8‐ANEPPS, which can increase its quantum yield when associated with the EV membrane (Figure 6C) (Adamo et al. 2021), (ii) performing immunoblotting analysis of the membrane protein H+‐ ATPase, characteristic of nanoalgosome bilayers (Figure 6D) and (ii) carrying out anti‐α‐tubulin immunoblotting analysis as a negative control to evaluate potential intracellular contamination (Figure 6E).

**FIGURE 7:**
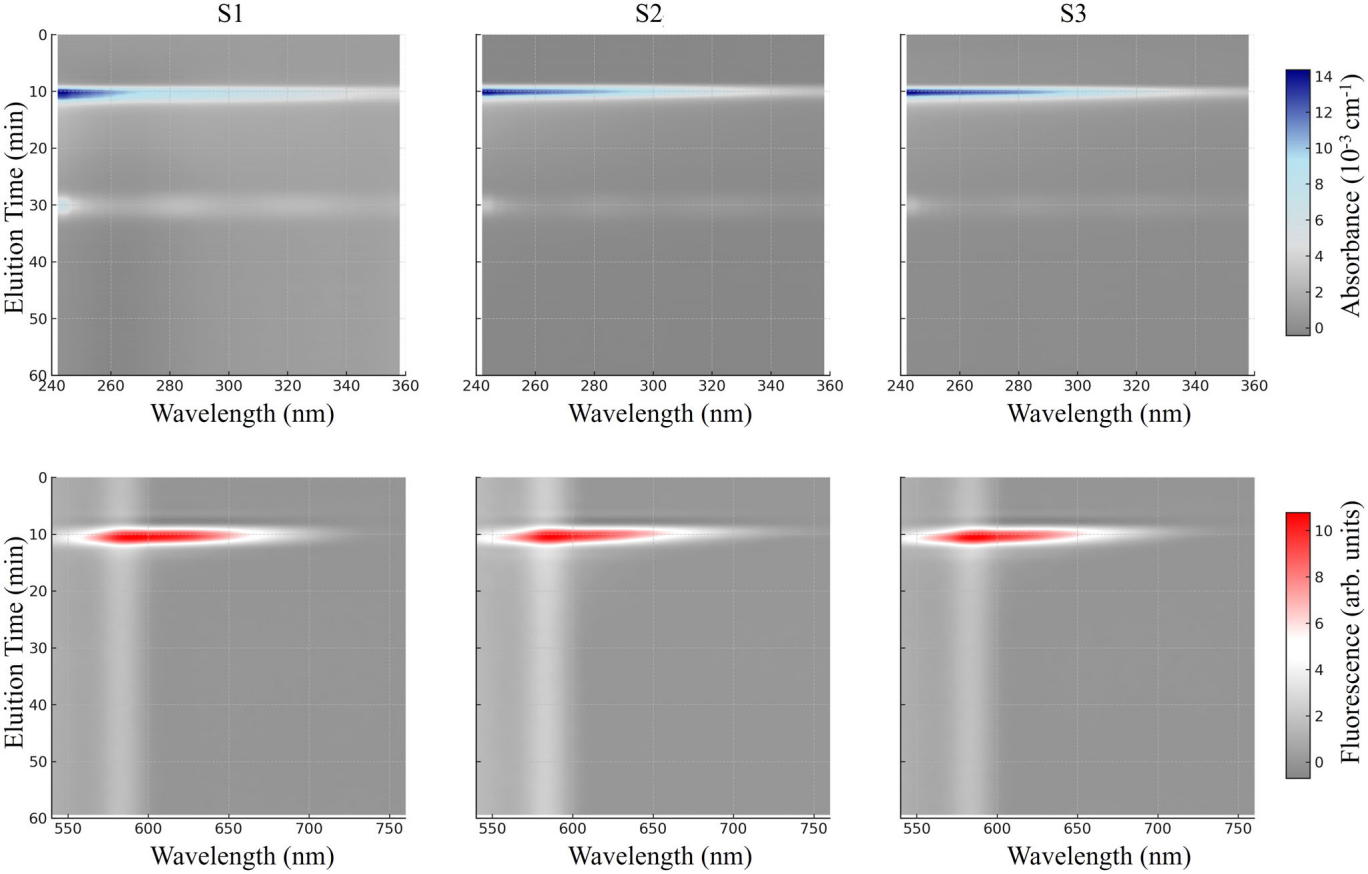
Chromatograms for 3 independent batches (s1, s2, s3). Upper panels: Optical density (absorbance). Lower panels: Photoluminescence (fluorescence) with excitation at 488 nm.

**FIGURE 8:**
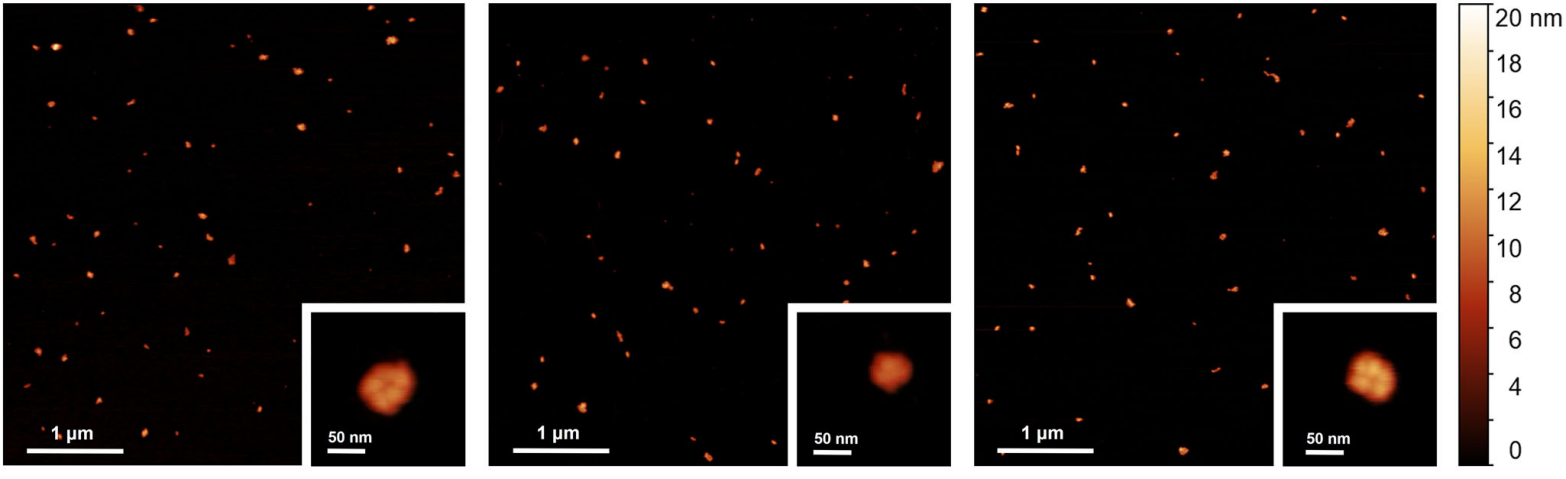
AFM images for 3 independent batches s1, s2 and s3; in the insets a representative object imaged at higher resolution.

*Size exclusion chromatography*. After the TFF cycles, each sample was injected into a column for size inclusion chromatography (SEC) to refine EV purification if necessary. The HPLC system is equipped with a diode array detector (DAD) to measure Optical Density (OD) (Figure 7 upper panels) and with a 3D fluorescence detector (Figure 7 upper panels). EV samples labeled with Di‐8‐ANEPPS dye elute at about 10 mL as reported by the combined absorbance and fluorescence signal. The OD plot evidence another residual population of small objects with no lipid content eluted at about 30 minutes (likely small proteins). In summary, the clean chromatograms confirm the reliability and efficacy of the sequential TFF procedure. *Atomic Force Microscopy*. The quality of EV purification is further assessed by AFM images of the three samples (Figure 8). The measurements were performed in air, after the samples were gently dried, to elicit any spurious object that may be present in the samples. The AFM images confirm that the EV samples are clean and homogeneous, with a negligible presence of smaller objects, as noted in the SEC experiments (Figure 7).

## 4 Conclusions

In this study, we developed and optimized a continuous scalable process based on TFF for the downstream isolation and purification of small extracellular vesicles (nanoalgosomes) derived from the marine microalga *Tetraselmis chuii*. By integrating upstream cultivation under controlled environmental conditions with a differential multistage TFF system, we demonstrated showed a robust and reproducible workflow that could support the development of industrial‐scale and GMP‐compliant production processes for small EVs. Through detailed theoretical and experimental analysis of key TFF parameters, including crossflow rate, shear stress, transmembrane pressure, and membrane fouling, we identified optimal operating conditions that maximize EV yield while preserving vesicle integrity. The system operates in the laminar flow regime, mitigating shear damage and ensuring product quality. Biochemical and biophysical characterizations in independent batches confirmed the purity, stability, and homogeneity of the final product, according to the MISEV2023 guidelines. Overall, our work contributes to a comprehensive and standardized platform for the large‐scale isolation of microalgal EVs. The process is sustainable, reproducible, under sterile conditions and compatible with further scale‐out or adaptation to other biological sources, paving the way for their exploitation in biomanufacturing, cosmetics, nutraceuticals, and nanomedicine. Future efforts will focus on integrating real‐time monitoring tools and automated control of key process variables, further improving scalability and batch‐to‐batch consistency.

## Author Contributions

**Angela Paterna**: conceptualization, investigation, methodology, writing – original draft, writing – review and editing. **Estella Rao**: investigation, methodology, data curation, writing – review and editing. **Paola Gargano**: investigation, writing – review and editing. **Sabrina Picciotto**: investigation, writing – review and editing. **Giorgia Adamo**: investigation, writing – review and editing. **Samuele Raccosta**: investigation, data curation, writing – review and editing. **Antonella Bongiovanni**: conceptualization, project administration, supervision, writing – review and editing. **Mauro Manno**: conceptualization, project administration, supervision, writing – original draft, writing – review and editing.

## Funding

This work was partially supported by the following projects: Protein Loaded extracellular vesicles As Next generation Therapeutics (PLANT, Project code 2022TF4BKK), funded by the European Union‐NextGenerationEU under the National Recovery and Resilience Plan (NRRP), concession Decree No. 104 13 of 2 February 2022 adopted by the Italian Ministry of University and Research, Mission 4, Component 2, Investiment 1.1; Programma Nazionale di Ricerca e Progetti di Interesse Nazionale (PRIN); Biomimetic Organotropic Wetsuit (BOW), funded by the European Union's Horizon 2020 research and innovation program, under grant agreements No 952183; European Brain ReseArch INfrastructureS‐Italy (EBRAINS‐ Italy, Project code IR0000011), funded by the European Union—NextGenerationEU under the National Recovery and Resilience Plan (NRRP), concession Decree No. 117 of 21 June 2022 adopted by the Italian Ministry of University and Research, Mission 4, Component 2, Investiment 3.1.

## Conflicts of Interest

The authors Antonella Bongiovanni and Mauro Manno declare the following financial competing interests: they have filed the patent (PCT/EP2020/086622) related to the microalgal‐derived extracellular vesicles described here and are co‐founders of EVEBiofactory srl., of which Antonella Bongiovanni is the CEO and Mauro Manno is a member of the Administrative Board. The remaining authors declare no conflicts of interests.

## Supporting information




**Supporting Information**: jex270185‐sup‐0001‐SuppMat.docx

## Data Availability

Data will be made available on corresponding author upon reasonable request.
