# Supplementary material for: Scalable and Controlled Production of Microalgae‐derived Extracellular Vesicles Using a Differential Tangential Flow Filtration Protocol
Source: J Extracell Biol. 2026 Sep 11;5(9):e70185. doi: 10.1002/jex2.70185 (PMC13563171; doi:10.1002/jex2.70185)
Supplement: Supplementary file 1 [file JEX2-5-e70185-s001.docx]

**---- Supplementary material ----**

**Scalable and controlled production of microalgae-derived extracellular vesicles using a differential tangential flow filtration protocol**

Angela Paterna^a,∗^, Estella Rao^a^, Paola Gargano^a,b,c^, Sabrina Picciotto^a,b^, Giorgia Adamo^b^, Samuele Raccosta^a^, Antonella Bongiovanni^b,c^, Mauro Manno^a,c,∗^

^a^ National Research Council of Italy (CNR), Cell-Tech HUB and Institute of Biophysics (IBF), via Ugo La Malfa 153, Palermo, 90146, Italy

^b^ National Research Council of Italy (CNR), Cell-Tech HUB and Institute for Biomedical Research and Innovation (IRIB), via Ugo La Malfa 153, Palermo, 90146, Italy

^c^ EVE Biofactory s.r.l., via G.M. Puglia 21, Palermo, 90138, Italy

**Supplementary Note 1**

Tangential flow filtration is a gentle filtration technique based on tangential feed flow parallel to the membrane surface of the starting solution; the transmembrane pressure (TMP) generated drives the components smaller than the pore size of the membrane to pass through the filter. In this section, we briefly review the general principles of TFF and outline an empirical framework, grounded in the literature, to elucidate its operational behavior in practical contexts.

*Flow through the pipe channel.* The **crossflow flux** also known as the feed flux or recirculate flux *J_F_* is defined as the volumetric flow rate *Q_F_* through the channel per unit area *A*; when the channel is made by *N* pipes of diameter *D*, as in the case of hollow fibers, the area is just *A = Nπ(D/2)^2^*:

$J_{F}=Q_{F}[N\pi(D/2)^{2}]^{-1}$	(1)

In the case of steady laminar flow, that is, a flow with fluid velocity only along the pipe axis *z* (without turbulence), the velocity variation *v(r)* in the radial direction *r* can be considered constant over the pipe axis, while the pressure *P* is constant in the radial direction. With the latter assumptions, one may derive from the general Navier-Stokes equations the so-called Hagen-Poiseuille equation [1,2]:

$\frac{1}{r}\frac{d}{dr}\left( r\frac{dv(r)}{dr} \right)=\frac{1}{\eta}\frac{dP}{dz}$	(2)

Where *η* is the fluid viscosity. The latter equation can be easily solved for a tube of length *L*, considering that the pressure gradient is constant and related to the pressure difference between the two ends of the pipe *∆P_F_ = P_feed_−P_retentate_* (that in TFF systems is the pressure at the feed and retentate side): $\frac{d}{dz}P=-L^{-1}\Delta P_{F}$	. It is straightforward to derive the typical parabolic shape of the fluid velocity $v(r)=\Delta P_{F}[4\eta L]^{-1}[(D/2)^{2}-r^{2}]$, and to calculate the volumetric flow rate $Q_{F}=N\int_{0}^{D/2}2\pi rv(r)dr$ and the shear rate $\gamma(r)=\frac{dv(r)}{dr}$. As a result, one obtains that the flux (*J_F_*) is equal to the average fluid velocity (*v_avg_*) and it is proportional to the pressure difference:

$J_{F}=\frac{D^{2}}{32\eta L}\Delta P_{F}$	(3)

In TFF systems, cross-flow through pipes is affected by the simultaneous existence of a transmembrane flow; therefore, it can be expected that a higher pressure difference at the end of the pipe is required to maintain a certain flux, and this is indeed the actual occurrence in our process, where $J_{F}<D^{2}(32\eta L)^{-1}\Delta P_{F}$.

Also, the **shear rate** at the membrane wall, *γ(D/2)* is proportional to the flux and the flow rate, as well as the corresponding **shear stress**, *σ=ηγ(D/2)*:

$\sigma=-\frac{8\eta}{D}J_{F}$	(4)

Therefore, in TFF applications it is important to limit the flow rate when the sensitivity of the bioproducts to shear stress may affect its yield and the quality of the final product. To determine or predict the flux regime, one may consider the balance between inertial forces, such as the hydrodynamic pressure in a fluid of density ρ ($\sim\rho J_{F}^{2}$), and viscous forces, such as the shear stress in the membrane wall ($\sim\eta J_{F}D^{-1}$). The ratio between these forces is expressed by the Reynolds number *Re*, which was originally introduced to rearrange the Navier-Stokes equations in terms of dimensionless quantities [1]:

$Re=\frac{\rho J_{F}D}{\eta}$	(5)

The Reynolds number is commonly used to predict a **turbulent flow regime**, assuming a laminar flow regime when *Re ≤ 2300* and a turbulent flow regime when *Re> 2900*. In TFF systems, we can associate the Reynolds number with the operational volumetric flow rate Q_F_: *Re = Q_F_/Q_0_*, where *Q_0_ = NπDη(4ρ)^−1^* is a characteristic flow rate depending upon the TFF fibers and the fluid properties. In Table 1, we report these parameters for our setup to indicate that our system operates under the conditions of the laminar flow regime.

*Flow through the filter membrane*. Along with the tangential crossflow, in TFF systems the solution is selectively driven through the filtration membrane. This so-called **permeate flux** *J_P_* is defined as the volumetric flow rate Q*_P_* through the membrane per unit area *Σ_m_*.

$J_{P}=Q_{P}\Sigma_{m}^{-1}\equiv LMH$	(6)

The permeate flux is indicated as LMH in technical literature since it is expressed in units of liters per hour per square meter (Lm^−2^h^−1^). If the membrane were made of *n* cylindrical pores with diameter, equal to the size of the filter and length *t*, equal to the thickness of the membrane, and we assume a steady laminar flow, we could write an expression analogous to the one derived from the Hagen- Poiseuille equation:

$J_{P}=\frac{d^{2}\epsilon_{s}}{32\eta t}\Delta P_{TMP}$	(7)

Where $\epsilon_{s}=n\pi(d/2)^{2}\Sigma^{-1}$ is the porosity of the membrane surface and ∆*P_TMP_*, also known as TMP in the technical literature, is the **transmembrane pressure**. In laboratory practice, under the assumption that the pressure inside the flow channel is the average between the pressures at the two ends (feed and retentate pressures), which is of course an approximation, this pressure difference is calculated as follows:

$\Delta P_{TMP}=\frac{P_{feed}+P_{retentate}}{2}-P_{permeate}\equiv TMP$	(8)

A former version of expression 7, also known as Darcy’s law, was originally (in the XIX century) derived to describe the flow through porous media and was then continuously improved by introducing the tortuosity of the path through the membrane or more complex correction depending upon the materials and the consistency of the filter membrane. The basic concept is that the flux through the membrane is proportional to the transmembrane pressure with a coefficient called membrane permeability [3]. For instance, the permeability of the water can be assessed by measuring the permeate flux *J_P_* as a function of the transmembrane pressure ∆*P_TMP_*. In particular, **normalized water permeability** (*NWP*) is a parameter that is often used to determine the efficacy of the cleaning process and the integrity of the filter. *NWP* is measured after cleaning and is compared to the value measured at membrane installation. In order to be independent of the operating temperature NWP is defined using a correction factor (*TCF*) that reports the viscosity of the water at a certain temperature *η(T)* to the viscosity of the water at 20°C:

$NWP=\frac{J_{P}}{\Delta P_{TMP}}\frac{\eta(T)}{\eta(20)} \left[ NWP=\frac{LMH\times TFC}{TMP} \right]$	(9)

*Mass transfer through the membrane*. The purpose of TFF is to transfer across the membrane the particles of size smaller than the membrane cutoff. In the case of laminar flux, the particles move along the tangential flow direction and at the same time undergo diffusion along the radial direction (Figure 1) [4]. As a consequence of the porous membrane walls, the particle con centration in the membrane wall (*c _wall_*) is different from the concentration in the bulk solution (*c_bulk_*) and from the concentration across the membrane, that is, the permeate concentration (*c_perm_*) [5]. In the so-called stagnant film model (steady laminar flow with negligible diffusion in the tangential direction), one may write a mass continuity equation for the concentration in the radial direction c(r) of particles of diffusion coefficient *D_p_* [6]:

$J_{P}c(r)=J_{P}c_{perm}-D_{p}\frac{dc(r)}{dr}$	(10)

The latter expression is easily solved with the boundary conditions that the concentration is equal to *c_wall_* at the membrane and to *c_bulk_* at the distance *δ* from the membrane walls. This parameter marks the thickness of this region of higher concentration, called the concentration polarization boundary layer. The concentration difference at the layer boundary reads:

$c_{wall}-c_{bulk}=(c_{bulk}-c_{perm})[e^{{J_{P}}/\kappa}-1]$	(11)

where *κ=D p/δ* is the mass transfer coefficient. This concentration polarization determines an additional pressure ∆Π that opposes the permeate flux within the boundary layer. At small concentrations, we may approximate this equivalent osmotic pressure, associated with the concentration polarisation in the boundary layer, as linearly proportional to the concentration difference *(∆Π∼ c_wall_ − c_bulk_*) and obtain (neglecting permeate concentration):

$\Delta\Pi=\pi_{0}[e^{{J_{P}}/\kappa}-1]$	(12)

Where π_0_ = c_bulk_ RT, being R the gas constant and T the temperature. One may consider that the transmembrane pressure operates against a resistance due to the membrane R*_m_*, that is the reciprocal of the permeability, and a resistance due to the boundary layer R*_bl_*:

$\Delta P_{TMP}=\eta(R_{m}+R_{bl})J_{P}$	(13)

$R_{m}=\frac{32t_{eff}}{d^{2}\epsilon_{s}}$	(14)

$R_{bl}=\frac{\pi_{0}}{\eta\kappa}\frac{e^{{J_{P}}/\kappa}-1}{{J_{P}}/\kappa}$	(15)

At low flux values, the main resistance is due to the membrane (linear regime), while at higher flux values the flow is affected by the boundary layer (polarisation regime) [3, 7]. The crowding of particles at the membrane walls may cause an obstacle to the permeate flux, the **membrane fouling**. This may be due to an excess of surface polarisation due to small particles that create a so-called gel layer; in the worst cases, it may also be due to large particles that block the channel and form a cake layer [2]. Membrane fouling is an important variable that should be evaluated during a TFF process assessment not only in terms of membrane lifetime but also in terms of product recovery, filtration rate, and flux decline. Indeed, the fouling operates as an additional resistance, causing the permeate flux to become independent of the transmembrane pressure as in the solid curve of Figure 2 [8]:

$\Delta P_{TMP}=(R_{m}+R_{bl}+R_{f})\eta J_{P}$	(16)

At high permeate flux, fouling becomes relevant, rate- limiting and it affects the transmembrane pressure in a non-stationary way [7,9]. Below a given threshold flux *J_1_*, when the fouling is mainly reversible [2,7] , we model this effect as an additional fouling resistance by using the following empirical expression:

$R_{f}=\pi_{1}\eta^{-1}(J_{1}-J_{P})^{-1}$	(17)

which remains useful and meaningful only when *J_P_* < *J_1_*(Figure 2). It is important to mitigate or avoid membrane fouling in order to improve filtration efficiency. A direct method to remove fouling could be to induce a turbulent flow regime and thus to reduce the concentration boundary layer. Other methods include periodic back wash cleaning during the filtration process and not only for cleaning and recovery [10]. Here, we adopt a different strategy that is to limit the flux and transmembrane pressure to the first polarisation regime, avoiding both the gel layer formation and the turbulence. In this manner, we have the highest possible flux at low Reynolds number, that is, in the laminar flow regime, avoiding a large shear stress that may affect our products. This is relevant not only for the isolation of extracellular vesicles itself, but also for the clarification step and to preserve the integrity of the cell sources, since we demonstrated the possibility of recycling the cell culture for the subsequent production of EV [11].

References:

[1] A. G. Mailer, P. S. Clegg, P. N. Pusey, Particle sizing by dynamic light scattering: non-linear cumulant analysis., Journal of physics. Condensed matter : an Institute of Physics journal 27 (14) (2015) 145102. doi:10.1088/0953-8984/27/14/145102.

[2] L. D. Landau, E. M. Lifshitz, Fluid Mechanics, Pergamon Press, Oxford, New York, 1987.

[3] H. Lutz, 2.06 - ultrafiltration: Fundamentals and engineering, in: E. Drioli, L. Giorno (Eds.), Comprehensive Membrane Science and Engineering, Elsevier, Oxford, 2010, pp. 115–139. doi:10.1016/B978-0-08-093250- 7.00037-2.

[4] R. Field, 2.07 - fundamentals of cross-flow microfiltration, in: E. Drioli, L. Giorno (Eds.), Comprehensive Membrane Science and Engineering, Elsevier, Oxford, 2010, pp. 141–153. doi:10.1016/B978-0-08-093250- 7.00021-9.

[5] E. S. Asmolov, The inertial lift on a spherical particle in a plane Poiseuille flow at large channel Reynolds number, Journal of Fluid Mechanics 381 (1999) 63–87. doi:10.1017/S0022112098003474.

[6] A. L. Zydney, Stagnant film model for concentration polarization in membrane systems, Journal of Membrane Science 130 (1) (1997) 275–281. doi:10.1016/S0376- 7388(97)00006-9. 16

[7] J. Wijmans, S. Nakao, J. Van Den Berg, F. Troelstra, C. Smolders, Hydrodynamic resistance of concentration polarization boundary layers in ultrafiltration, Journal of Membrane Science 22 (1) (1985) 117–135. doi:10.1016/S0376- 7388(00)80534-7.

[8] R. Field, D. Wu, J. A. Howell, B. B. Gupta, Critical flux concept for microfiltration fouling, Journal of Membrane Science 100 (1995) 259–272. doi:10.1016/0376-7388(94)00265-Z.

[9] A. Y . Kirschner, Y .-H. Cheng, D. R. Paul, R. W. Field, B. D. Freeman, Fouling mechanisms in constant flux crossflow ultrafiltration, Journal of Membrane Science 574 (2019) 65–75. doi:10.1016/j.memsci.2018.12.001.

[10] P. Romann, P. Giller, A. Sibilia, C. Herwig, A. L. Zydney, A. Perilleux, J. Souquet, J.-M. Bielser, T. K. Villiger, Co-current filtrate flow in tff perfusion processes: Decoupling transmembrane pressure from crossflow to improve product sieving, Biotechnology and Bioengineering 121 (2) (2024) 640–654. doi:10.1002/bit.28589

[11] A. Paterna, E. Rao, G. Adamo, S. Raccosta, S. Picciotto, D. Romancino, R. Noto, N. Touzet, A. Bongiovanni, M. Manno, Isolation of Extracellular Vesicles from Microalgae: A Renewable and Scalable Bioprocess, Frontiers in Bioengineering and Biotechnology 10 (March) (2022) 836747. doi:10.3389/fbioe.2022.836747.

Figure 1: Schematic representation of TFF systems, displaying the tangential and normal fluxes (**J***_F_* and **J***_P_*, respectively), the thickness δ of the concentration polarisation boundary layer, the relevant boundary layer concentrations for a solute in the flow **c***_bulk_*, **c***_wall_* and **c***_perm_*, that are the bulk concentration, the concentration at the membrane wall and the concentration of the permeate solution, respectively.

Figure 2: Permeate flux **J***_P_* versus transmembrane pressure ∆**P**_TMP_ forgiven values of membrane resistance R*_m_*, boundary layer resistance R_bl_ and fouling resistance R*_f_* ; (i) dotted line: R*_m_*= 0.25 mbar (L m^−2^ h^−1^)^−1^, R*_bl_*=0, R*_f_* =0; (ii) dashed line: R*_m_* as in the previous curve, R*_f_* =0, R*_bl_* determined assuming a bulk osmotic pressure *π*_0_= 24 mbar and a mass transfer coefficient κ= 360 L m^−2^ h^−1^ = 100 μm s^−1^; (iii) solid line: R*_m_* and R*_f_* as in the previous curve, R*_f_* determined assum-ing a threshold flow *J*_1_= 720 L m^−2^ h^−1^ = 200 μm s^−1^ and a pressure *π*_1_ = 24 mbar.
